# Effects of caffeine on the detection of ischemia in patients undergoing adenosine stress cardiovascular magnetic resonance imaging

**DOI:** 10.1186/s12968-017-0412-0

**Published:** 2017-12-18

**Authors:** Simon Greulich, Philipp Kaesemann, Andreas Seitz, Stefan Birkmeier, Eed Abu-Zaid, Francesco Vecchio, Udo Sechtem, Heiko Mahrholdt

**Affiliations:** 1 Division of Cardiology, Robert-Bosch-Medical Center Stuttgart, Auerbachstrasse 110, 70376 Stuttgart, Germany; 2 Department of Cardiology and Cardiovascular Diseases, University Hospital Tübingen, Tübingen, Germany; 3 Division of Cardiology, Kliniken Dr. Müller, Munich, Germany

**Keywords:** Caffeine, Ischemia, Adenosine, Stress, CMR

## Abstract

**Background:**

Adenosine stress cardiovascular magnetic resonance (CMR) can detect significant coronary artery stenoses with high diagnostic accuracy. Caffeine is a nonselective competitive inhibitor of adenosine2A-receptors, which might hamper the vasodilator effect of adenosine stress, potentially yielding false-negative results. Much controversy exists about the influence of caffeine on adenosine myocardial perfusion imaging. Our study sought to investigate the effects of caffeine on ischemia detection in patients with suspected or known coronary artery disease (CAD) undergoing adenosine stress CMR.

**Methods:**

Thirty patients with evidence of myocardial ischemia on caffeine-naïve adenosine stress CMR were prospectively enrolled and underwent repeat adenosine stress CMR after intake of 200 mg caffeine. Both CMR exams were then compared for evaluation of ischemic burden.

**Results:**

Despite intake of caffeine, no conversion of a positive to a negative stress study occurred on a per patient basis. Although we found significant lower ischemic burden in CMR exams with caffeine compared to caffeine-naïve CMR exams, absolute differences varied only slightly (1 segment based on a 16-segment model, 3 segments on a 60-segment model, and 1 ml in total ischemic myocardial volume, *p* < 0.001 each). Moreover, no relevant ischemia (≥2 segments in a 16-segment model) was missed by prior ingestion of caffeine.

**Conclusions:**

Although differences were small and no relevant myocardial ischemia had been missed, prior consumption of caffeine led to significant reduction of ischemic burden, and might lower the high diagnostic and prognostic value of adenosine stress CMR. Therefore, we suggest that patients should still refrain from caffeine prior adenosine stress CMR tests.

## Condensed abstract

We investigated the effect of caffeine on adenosine stress perfusion CMR ischemia detection in patients with suspected or known coronary artery disease.

Thirty patients with evidence of myocardial ischemia on caffeine-naïve adenosine stress CMR underwent repeat adenosine stress CMR after intake of caffeine. Both exams were compared for evaluation of ischemic burden.

Although differences were small and no relevant myocardial ischemia had been missed, prior consumption of caffeine led to significant reduction of ischemic burden and might lower the high diagnostic value of adenosine stress CMR. Therefore, we suggest that patients should still refrain from caffeine prior adenosine stress CMR.

## Background

Caffeine is a component of many beverages and foods including coffee, tea, soft drinks, energy drinks, and chocolate [1]. Coffee is routinely consumed by 80 % of the population in the United States [2]. Stress cardiovascular magnetic resonance (CMR) perfusion tests with adenosine are increasingly performed and have proven to be of high diagnostic accuracy for the non-invasive evaluation of myocardial ischemia (ischemic burden) in patients with significant coronary artery stenoses by inducing coronary hyperemia via stimulation of the adenosine2A-receptor [3–6].

Current imaging guidelines recommend the avoidance of caffeine intake for at least 12–24 h in patients undergoing adenosine stress tests [7, 8], since caffeine 1) is a nonselective competitive inhibitor of adenosine2A-receptors, which might hamper the vasodilator effect of adenosine, and 2) increases sympathetic activity which might lead to capillary de-recruitment, resulting in decreased myocardial perfusion reserve [9, 10], with both effects potentially yielding false-negative stress results.

However, recommendations to refrain from caffeine prior vasodilator stress imaging are based largely on several false-negative dipyridamole (another vasodilator agent by stimulating adenosine2A receptor) myocardial perfusion studies in the presence of caffeine [11, 12]. Furthermore, there is conflicting data since other studies suggest a negligible effect of caffeine on the results of myocardial perfusion imaging with adenosine on single-photon emission computed tomography (SPECT) [13, 14].

Investigating the influence of caffeine on the diagnostic performance of an adenosine CMR stress test is of high clinical importance since nowadays performing institutions will commonly face patients with caffeine consumption prior 24 h of the exam although these were instructed to avoid any caffeine intake. Normally, the adenosine stress test is then rescheduled to another day resulting in: 1) inconvenience for both patient and institution, and even worse 2) delay of the patient’s diagnosis.

Consequently, this study was designed to determine the effects of caffeine on the detection of ischemia by performing a head-to-head comparison of a caffeine-naïve CMR scan (with evidence of myocardial ischemia) to a repeat adenosine stress CMR scan after defined consumption of caffeine.

## Methods

### Patient population

Patients with known or suspected coronary artery disease (CAD) referred for stress CMR at our institution were asked before stress CMR if they would like to participate in this study and were prospectively enrolled. Definite inclusion criteria were: 1) reversible myocardial ischemia (≥2 segments of the 16-segment model) [15] in their initial CMR with caffeine abstention, and 2) return for repeat adenosine stress CMR with defined intake of 2 cups of coffee with each patient getting the same sort and size of 2 capsules of coffee with an overall caffeine content of 200 mg [16] one hour prior the exam to allow plasma caffeine level to reach its maximum [17, 18], and 3) no coronary intervention and no change in clinical status or medication between initial and follow-up CMR. For each visit, patients were asked to refrain from caffeine and anti-anginal medication 24 h before CMR. Patient daily caffeine habits were reported. Participants provided a blood sample for measurement of serum caffeine levels both at initial and repeat CMR exam. 200 mg caffeine is known to inhibit the hemodynamic response to intravenous adenosine [19]. An immunoassay technique (Bioscientia, Ingelheim, Germany) was used to measure caffeine levels. All patients gave written informed consent, and the study has been approved by the ethics committee of the University of Tuebingen, Germany.

### CMR protocol

Details of the adenosine stress CMR protocol have been reported previously [20]. Electrocardiom-gated CMR was performed in breath-hold using a 1.5 T (MAGNETOM Aera, Siemens Healthineers, Erlangen, Germany) in line with current recommendations [21]. In brief, balanced steady-state free-precession cine images for assessment of left ventricular (LV) function were acquired in multiple short-axis and three long-axis views. Adenosine (140 μg·kg^−1^·min^−1^) gadolinium (0.07 mmol/kg gadopentetate) first-pass imaging for assessment of stress perfusion was performed in three short axis views (basal, mid, apical) covering 16 of the standard myocardial segments [15] using a saturation-recovery, single-shot, gradient-echo sequence [20]. Repeat first-pass images without adenosine 15 min later were performed for assessment of rest perfusion. Five minutes after rest perfusion late gadolinium enhancement (LGE) was performed using a segmented inversion-recovery technique.

### CMR analysis

Initial (caffeine-naïve) and repeat (after intake of caffeine) CMR scans were analyzed side by side by consensus of two experienced observers (S.G., P.K.) blinded to patient identity, clinical information, caffeine levels, status of caffeine intake, and coronary angiography results. A perfusion defect was defined as a visual regional dark area, that 1) persisted for >2 beats while other regions enhanced during the first-pass of contrast through the myocardium, and 2) involved the subendocardium [22, 23]. Dark rim artifact was not regarded as perfusion deficit using previously described criteria [24].

Beside dichotomous analysis (presence or absence of ischemia), the extent of myocardial perfusion defects (ischemic burden) was analyzed quantitatively by calculating the total number of ischemic segments based on the 1) established 16-segment model basis [15], and 2) 60-segment model basis with each of the three perfusion slices (basal, mid, apical) further divided into 20 segments per slice, and 3) total volume basis (ml) by the use of dedicated software (QMass, Medis, Leiden, the Netherlands). Significant CAD (≥70% stenosis) was confirmed by invasive coronary angiography in all 30 patients demonstrating myocardial ischemia on adenosine stress CMR.

Cine and LGE images were evaluated as described elsewhere [25]. In brief, endocardial and epicardial borders were outlined on the short axis cine images. Volumes and ejection fraction were derived by summation of epicardial and endocardial contours. The distribution of LGE was characterized as epicardial, intramural, transmural, or subendocardial [25].

### Statistical analysis

Absolute numbers and percentages were computed to describe the patient population. Normally distributed continuous variables were expressed as means (with standard deviation). Comparisons between groups were made using the Mann-Whitney U test or the Fisher’s exact test, as appropriate. *P*-values (two-tailed) of <0.05 were considered significant. Pearson correlation was used to assess the variation in ischemic myocardial segments according to serum caffeine concentration. All statistical analyses were performed using SPSS (version 22.0, International Business Machines, Armonk, New York, USA).

## Results

### Patient characteristics

**Fig. 1:**
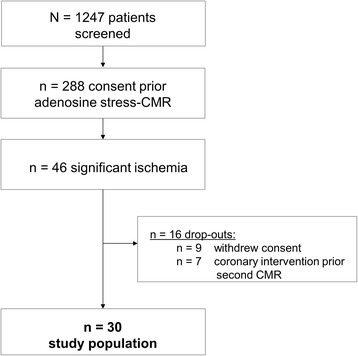
Flow chart demonstrating the study population

Of the *n* = 1247 screened patients, *n* = 288 gave informed consent to the study protocol prior adenosine stress CMR, see Fig. 1. Of these, *n* = 46 demonstrated significant myocardial ischemia, *n* = 16 were drop outs (*n* = 9 withdrew consent, *n* = 7 were revascularized before second stress CMR could be performed). The remaining *n* = 30 patients with significant myocardial ischemia returned for another CMR study with defined prior caffeine intake, see Table 1.

**Table 1:** Patient baseline characteristics

		*n* = 30
Age [yrs]		68 ± 8
Gender	Male	25 (83%)
Cardiovascular risk factors	Hypertension	24 (80%)
	Family history for CVD	15 (50%)
	Hyperlipidemia	25 (83%)
	Diabetes mellitus	10 (33%)
	Smoking	2 (6.7%)
Symptoms	None	3 (10%)
	Angina pectoris (AP)	16 (53.3%)
	Typical AP	13
	CCS I	–
	CCS II	8
	CCS III	5
	CCS IV	3
	Atypical AP	3
	Dyspnea	6 (20%)
	NYHA I	1
	NYHA II	4
	NYHA III	1
	NYHA IV	–
	Typ. AP + Dyspnea	5 (16.7%)
Medication	ASS	13 (43.3%)
	DAPT	7 (23.3%)
	ACE/ARB-Inhibitor	13 (43.3%)
	Betablockers	13 (43.3%)
	Statins	17 (56.7%)
	Nitrates/CCB	13 (43.3%)
	OAD	6 (20%)
ECG abnormalities		20 (66.7%)
Known CAD		14 (46.7%)
Prior CABG		4 (13.3%)
Prior MI/Myocardial scar		9 (30%)
Caffeine levels [mg/L]	baseline CMR	< 1
	follow-up CMR	4.6 ± 2.2
Daily caffeine consumption [cups]	coffee	3.0 ± 1.9
	tea	3.0 ± 1.5
Time between baseline and follow-up CMR [days]		13.9
CMR findings		
	LV-EF [%]	62.7 ± 8.4
	LV-EDV [ml]	132.3 ± 33.2
	LV-ESV [ml]	51.6 ± 24.0
	LA [cm^2^]	21.6 ± 4.0
	IVS [mm]	12.6 ± 2.4

All values are n (%), or mean ± SD, *CVD* cardiovascular disease, *ECG* electrocardiogram, *AP* angina pectoris, *ASS* acetylsalicylic acid, *DAPT* dual antiplatelet therapy, *ACE* angiotensin converting enzyme, *ARB* angiotensin receptor blockers, *CCB* calcium channel blocker, *OAD* oral antidiabetic drugs, *CAD* coronary artery disease, *CABG* coronary artery bypass graft, *MI* myocardial infarction, *CMR* cardiovascular magnetic resonance, *LV-EF* left-ventricular ejection fraction, *LV-EDV* left-ventricular end-diastolic volume, *LV-ESV* left ventricular end-systolic volume, *LA* left atrium, *IVS* interventricular septum

Patients were 68 ± 8 years of age, predominantly male (83%), and habitual caffeine consumers (mean daily consumption of 3 cups of tea or coffee, or both).

Almost one-half of the patients (47%) had known CAD, 30% showed ischemic LGE; all patients underwent two adenosine stress CMR exams. No patient had evidence of caffeine during the initial CMR (all serum caffeine levels <1 mg/L). However, elevated caffeine levels (4.6 ± 2.2 mg/L) were observed during the repeat CMR exam after caffeine intake. Mean duration was 2 weeks between initial (caffeine-naïve) and repeat (after caffeine intake) adenosine stress CMR.

### Myocardial perfusion defect by visual interpretation on a dichotomous basis

**Fig. 2:**
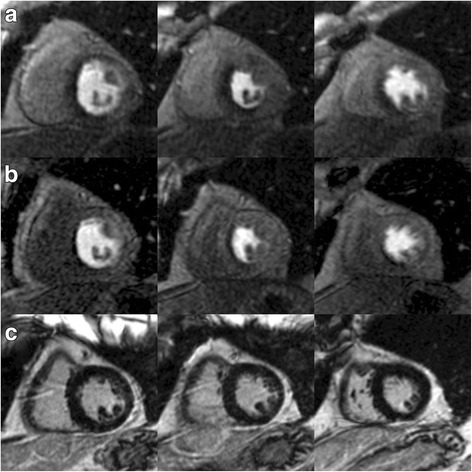
CMR Perfusion without vs. with caffeine. Adenosine stress cardiovascular magnetic resonance (CMR) images (basal, mid-ventricular and apical slices) without intake of caffeine (**a**) and the corresponding images after intake of 200 mg caffeine 1 h prior a repeat adenosine stress CMR (**b**) of a 85-year old male with known coronary artery disease (CAD) and typical angina pectoris demonstrating a perfusion defect in septal, inferoseptal and inferior segments (9 out of 16 segments) which seems to be larger without caffeine (**a**) than after intake of caffeine (**b**). LGE images revealed no late gadolinium enhancement (LGE) (**c**). Coronary angiography demonstrated severe stenosis of a) the proximal part of the left anterior descending coronary artery and b) of the mid segment of the right coronary artery, matching the results of (both) adenosine CMR stress perfusions

Dichotomous interpretation identified ischemic burden in all caffeine-naïve adenosine CMR stress tests and all follow-up adenosine CMR stress tests with prior caffeine consumption. Thus, no ischemia was missed visually despite the intake of caffeine. However, ischemic burden seems to be visually reduced after caffeine intake, Fig. 2.

### Hemodynamic variables and symptoms by caffeine status

Heart rate, systolic and diastolic blood pressure, and symptoms did not differ significantly between caffeine-naïve adenosine CMR stress tests and the follow-up adenosine CMR stress tests with prior caffeine consumption, all *p*-values >0.05, also see Table 2.

**Table 2:** Hemodynamic variables and symptoms

	W/o caffeine		W caffeine		*p*-values
	Rest	Adenosine	Rest	Adenosine	
Heart rate [1/min]	66.9 ± 9.4	84.7 ± 11.4	71.3 ± 11.3	83.7 ± 8.1	rest: *p* = 0.23
					adenosine: *p* = 0.73
Systolic blood pressure [mmHg]	154 ± 23	148 ± 22	154 ± 25	152 ± 24	rest: *p* = 0.79
					adenosine: *p* = 0.35
Diastolic blood pressure [mmHg]	89 ± 13	88 ± 12	88 ± 9	87 ± 9	rest: *p* = 0.73
					adenosine: *p* = 0.76
Symptoms of adenosine:					
Dyspnea		47%		43%	*p* = 0.32
Chest pain		28%		31%	*p* = 0.85

### Myocardial perfusion defect by segment and by total volume

The observation that ischemic burden tends to be reduced after caffeine intake could be confirmed by analysis in a 16-segment model: 7.9 ± 3.5 segments demonstrated myocardial ischemia without caffeine vs. 6.9 ± 3.5 segments with caffeine, *p* < 0.001. However, in one single patient no reduction was present, since two ischemic segments were present in the caffeine-naïve adenosine stress CMR, as well as after caffeine ingestion.

The more detailed 60-segment model revealed an even higher difference of ischemic segments between caffeine-naive vs. caffeine-ingested adenosine CMR stress scans: 18.6 ± 8.7 vs. 15.7 ± 8.7 segments, *p* < 0.001.

**Fig. 3:**
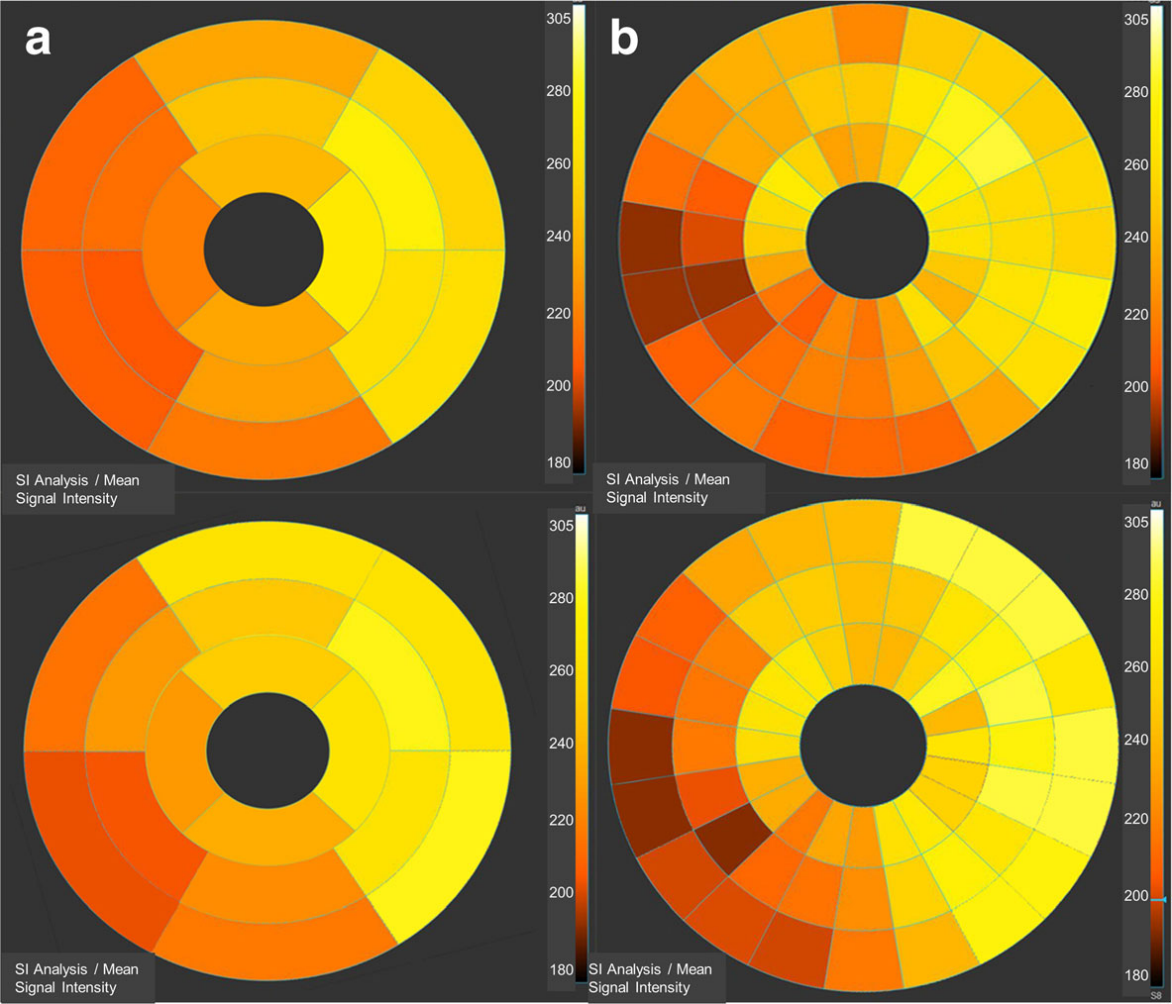
Ischemic burden on a segment model basis. Bulls-eye graphs representing the mean signal intensity in the same patient as in fig. 2 according to a 16-segment model (**a**) and a 60-segment model (**b**) in arbitrary units, with darker colors representing lower signal intensity values indicating impaired myocardial perfusion. Top row: Caffeine-naïve adenosine stress perfusion demonstrating a larger extent of ischemic burden compared to the adenosine stress CMR after intake of caffeine in the same patient, bottom row

**Fig. 4:**
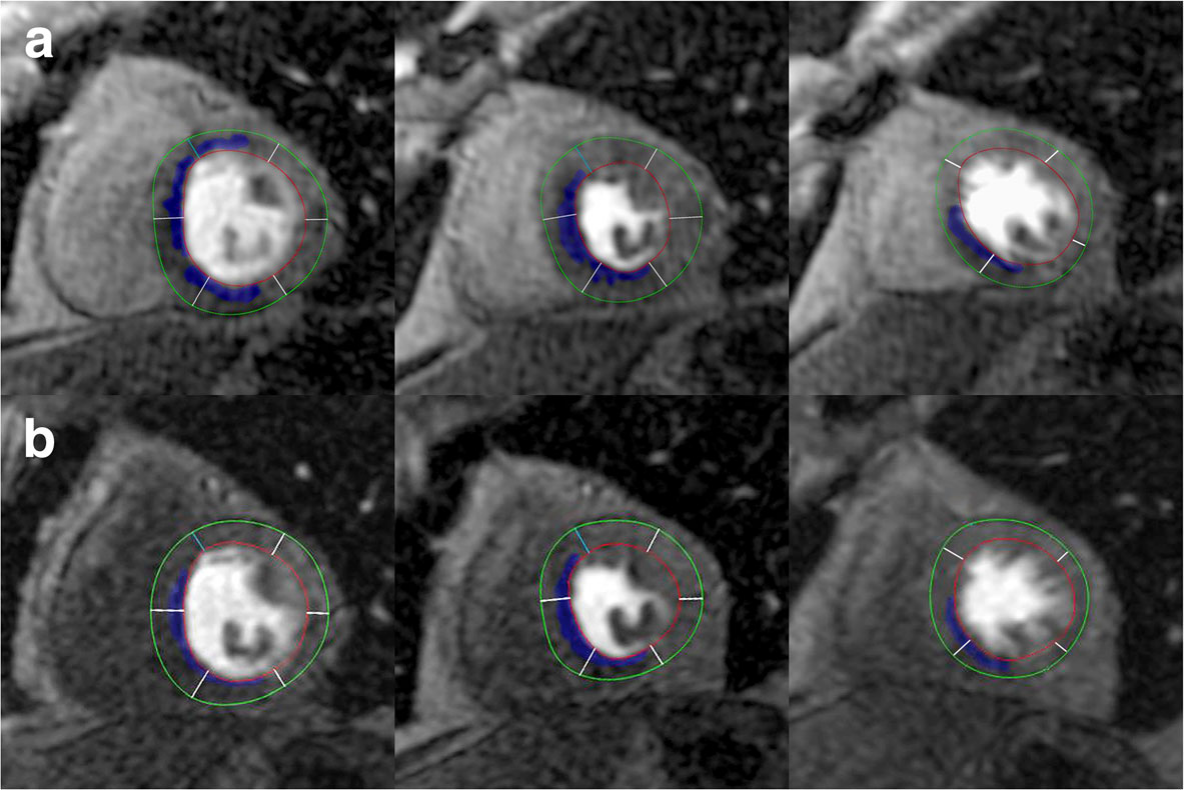
Absolute ischemic burden quantification. On the top row (**a**) absolute ischemic burden (in ml) is displayed without prior intake caffeine in the same patient than in Figs. 2 and 3. Bottom row (**b**) shows the corresponding perfusion slices after consumption of 200 mg caffeine one hour prior to the repeat scan. Similar to figs. 2 and 3, myocardial ischemic burden is reduced but still detectable in perfusion slices despite the influence of caffeine

Likewise, total ischemic volumes between CMR scans of caffeine-naïve vs. caffeine-exposed patients demonstrated significant differences: 4.2 ± 2.5 ml vs. 3.4 ± 2.4 ml, *p* < 0.001. Figures 2, 3 and 4 are images of the same patient illustrating lower myocardial ischemia on adenosine stress CMR after caffeine intake than on his caffeine-naïve scan based on different approaches: a) dichotomous, b) segmental, and c) total ischemic volume.

### Patient subgroups

Subgroup analysis revealed that patients with no evidence of LGE compared to patients with ischemic LGE demonstrated no major differences in the number of ischemic segments in their initial (caffeine-naïve) vs. their second (caffeine-ingested) CMR scan according to the 16-segment model, *p* = 0.89, the 60-segment model, *p* = 0.46, as well as for the quantified total ischemic volume, *p* = 0.37, see Table 3. Likewise, ischemic burden between initial and repeat CMR scan did not differ significantly in patients without a history of CAD vs. patients with known CAD based on the 16-segment model (*p* = 0.43), 60-segment model (*p* = 0.73), and total ischemic volume (*p* = 0.85). Furthermore, in patients with no previous coronary artery bypass graft (CABG) vs. patients with prior CABG, segments of ischemia in a 16-segment model, 60-segment model, and total ischemic volume demonstrated no significant differences between both CMR exams (*p* = 0.58, *p* = 0.18, *p* = 0.26, respectively). These results underline that caffeine itself seems to be the main driver of the reduced myocardial ischemia, independent from other conditions such as presence of myocardial scar, a history of known CAD or prior CABG.

**Table 3:** Subgroup analysis

	N	16-segments model			60-segments model			Ischemic volume [ml]		
		W/o caffeine	W caffeine	*p*-values*	W/o caffeine	W caffeine	*p*-values*	W/o caffeine	W caffeine	*p*-values*
No infarction	21	7.1 ± 3.5	6.1 ± 3.3	p = 0.89	15.8 ± 7	13.4 ± 7.1	p = 0.46	4.1 ± 2.2	3.5 ± 2.4	*p* = 0.37
Infarction^a^	9	9.8 ± 2.9	8.9 ± 3.3		25.1 ± 9.1	21 ± 10.1		4.4 ± 3.1	3.2 ± 2.5	
No CAD	14	8.3 ± 2.8	7.6 ± 3.8	*p* = 0.43	20.6 ± 9.5	17.3 ± 10.0	*p* = 0.73	4.4 ± 2.8	3.6 ± 3.0	*p* = 0.85
Known CAD	16	7.5 ± 4.3	6.3 ± 3		16.4 ± 7.4	13.9 ± 6.8		4.0 ± 2.1	3.1 ± 1.4	
No CABG	26	7.5 ± 3.5	6.6 ± 3.6	*p* = 0.58	18.2 ± 9.3	14.8 ± 8.7	*p* = 0.18	4.2 ± 2.7	3.3 ± 2.5	*p* = 0.26
CABG	4	10.8 ± 2.2	9.3 ± 1		21.0 ± 2.9	21.8 ± 5.9		4.2 ± 0.2	4.3 ± 1.1	

*= *p*-values for difference in segments/volume (w/o caffeine - w caffeine) between groups (e.g. no infarction vs. infarction)

^a^ = defined as ischemic type late gadolinium enhancement (LGE), other abbreviations see Table 1

### Caffeine levels and ischemic segments

Despite varying caffeine levels (4.6 ± 2.2 mg/L) after 200 mg caffeine with 60 min time to achieve maximum of plasma level [18], no correlation could be found between caffeine levels and the number of ischemic segments.

## Discussion

This is the first study evaluating the influence of caffeine on the ischemic burden by a two-exam adenosine stress CMR protocol including both a caffeine-naïve CMR scan and a repeat CMR scan after defined caffeine intake. Major findings are: 1) Despite intake of caffeine prior the repeat CMR exam, no conversion of an ischemic-positive to an ischemic-negative study could be observed in this high-risk population. 2) Although significant differences in the extent of ischemic burden were demonstrated between caffeine-naïve and post-caffeine CMR exams based on a 16-segment model, 60-segment model and total ischemic volume, differences were small in absolute terms, and no prognostic relevant myocardial ischemia (≥2 segments in a 16-segment model) was missed despite consumption of caffeine [26]. 3) A history of CAD seems to have no influence, since no significant differences could be observed for patients with prior myocardial infarction, known CAD or previous CABG vs. patients with no history of CAD. 4) No correlation could be found between serum caffeine levels and the number of ischemic segments.

### Patient characteristics

In total, *n* = 30 subjects were included in this study, Fig. 1; no patient had evidence of caffeine during the time of the initial CMR (all serum caffeine levels <1 mg/L). In contrast, increased caffeine levels 4.6 ± 2.2 mg/L during the second CMR exam in our study are within a range attenuating effects on the detection of ischemia since other studies suggest that a caffeine level of 2.0–2.9 mg/L should be the lower limit for a false-negative study [27, 28].

### Myocardial perfusion defect by visual interpretation on a dichotomous basis

Visual interpretation on a dichotomous basis (presence or absence of ischemia) revealed ischemia in both initial and repeat adenosine CMR scans. Therefore, despite prior caffeine intake, adenosine stress CMR still seems to be a valuable diagnostic tool for the detection of significant CAD. This is in line with previous studies suggesting a negligible effect of caffeine on the results of myocardial perfusion studies [13, 14]. Of note, on a per patient basis no relevant ischemia was missed despite the intake of caffeine. However, ischemic burden seems to be reduced after caffeine intake, Fig. 2.

### Myocardial perfusion defect by segment and total volume

Our observation that ischemic burden tends to be reduced after prior caffeine intake could be confirmed by quantitative analysis of the 16-segment model: 7.9 ± 3.5 segments showed myocardial ischemia without caffeine vs. 6.9 ± 3.5 segments after caffeine intake, *p* < 0.001. Therefore, despite not missing any relevant myocardial ischemia under caffeine, there seems to be attenuation of ischemic burden induced by the presence of caffeine. This effect might be in part explained by the caffeine dose of 200 mg, which is known to represent “significant” amount of caffeine [19]. In contrast, Zoghbi et al. [14] studied the effect of an 8-oz. cup of brewed caffeinated coffee (with a caffeine content varying from 25 mg to 240 mg) one hour before adenosine gated SPECT. Consequently, the latter study [14] demonstrated lower caffeine levels ranging from 3.1 ± 1.6 mg/L, whereas in the present study patients showed caffeine levels in the range of 4.6 ± 2.2 mg/L, suggesting a distinct effect of caffeine on coronary hyperemia. Our results are in line with a study from Namdar et al. [9]. They studied the effect of 200 mg caffeine (equivalent to our dose) on myocardial blood flow at rest and exercise in healthy volunteers at normoxia and during acute exposure to stimulated altitude by ^15^O–labeled H_2_O and positron emission tomography. They found that a dose of two cups of coffee (200 mg caffeine) significantly decreased exercise-induced myocardial blood flow at normoxia and at hypoxia, suggesting that exercise-induced hyperemic flow response may at least in part be antagonized by caffeine [9].

Most of our patients had ischemic burden comprising several myocardial segments, Figs. 2, 3 and 4. Data analysis revealed 7.9 ± 3.5 ischemic segments without caffeine vs. 6.9 ± 3.5 ischemic segments with caffeine, identifying our patients cohort as a subset of very-high-risk patients, since another group could show [29] that patients with >5 ischemic (of 16) segments had a risk of an adverse CAD event of approximately 14%/year. Although our results emphasize that on a 16-segment model basis the influence of caffeine leads to a decrease of ischemic burden by only 1 segment (7.9 ± 3.5 vs. 6.9 ± 3.5), these differences were significant (*p* < 0.001). One might argue that for patients further management, there won’t be a big difference if eight or seven myocardial segments were involved, postulated that these ischemic segments are supplied by the same coronary artery. However, studies suggest that the unadjusted hazard for CAD, death or myocardial infarction was elevated approximately by 1.2 for every segment with an ischemic perfusion defect compared to patients with a normal stress study who have an observed CAD event rate of approximately 1%/year [26, 30, 31]. Therefore, caffeine-induced effects on myocardial ischemia might mask not only patients’ accurate diagnosis but also his prognosis.

Interestingly, one of our patients had only 2 ischemic segments in his caffeine-naïve exam, which is considered as a threshold for moderate-severe myocardial ischemia [26], indicating adverse outcome which might warrant further invasive diagnosis by coronary angiography. In this case, despite intake of caffeine in the follow-up exam, the repeat scan still demonstrated 2 ischemic segments, pointing towards the hypothesis that no prognostic relevant myocardial ischemia is missed by prior caffeine intake in an adenosine stress CMR test. However, in cases in which only 2 of 16 myocardial segments are involved, prior caffeine might substantially increase the risk of a false-negative stress CMR or at least the probability to detect myocardial ischemia in just a single instead of two segments. At first sight, this might be a negligible difference. However, it is of clinical importance to detect the true extent of ischemic burden not only for diagnostic but also for prognostic purposes [26, 32], since patients with zero or just one ischemic segment can be safely deferred from revascularizations and show a favorable outcome on medical treatment that does not differ from those patients with normal CMR perfusion studies [32]. Based on our findings, we should expect, that in lower ischemic burden patients significant ischemia would be missed, affecting not only prognostic assessment but the diagnosis itself.

In the 60-segment model, we found an even higher difference in ischemic segments between caffeine-naive and caffeine adenosine CMR stress scans: 18.6 ± 8.7 vs. 15.7 ± 8.7 segments, *p* < 0.001. This illustrates that even in a very detailed model results are comparable to the results of the 16-segment model by showing slight, but significant differences in the absolute number of ischemic segments induced by prior caffeine consumption.

Likewise, total ischemic volume between caffeine-naïve and caffeine-consumed stress scans demonstrated slight but significant differences: 4.2 ± 2.5 ml vs. 3.4 ± 2.4 ml, p < 0.001, Figs. 2, 3 and 4, underlining a low but significant impact of caffeine on the extent of ischemic burden in adenosine stress CMR tests.

### Patient subgroups

Subgroup analysis revealed that patients with no LGE compared to patients with ischemic LGE demonstrated no major differences in the number of ischemic segments in their initial vs. their repeat CMR scan with regard to the 16-segment model, *p* = 0.89, the 60-segment model, *p* = 0.46, as well as for the total quantified ischemic volume, *p* = 0.37, Table 3. This is of importance since one might argue that LGE in patients might interfere with the potential extent of myocardial ischemia especially in terms of fixed perfusion defects. Similar results could be observed for the ischemic burden between the initial and repeat CMR scan in patients without a history of CAD vs. patients with known CAD with regard to the 16-segment model (*p* = 0.43), 60-segment model (*p* = 0.73), and total ischemic volume (*p* = 0.85). Likewise, in patients with no previous coronary artery bypass graft (CABG) vs. patients with prior CABG, segments of ischemia in a 16-segment model, 60-segment model, and total ischemic volume demonstrated no significant differences between both CMR exams (*p* = 0.58, *p* = 0.18, *p* = 0.26, respectively). These results underline that the presence of caffeine itself seems to be the main driver of a reduced ischemic burden, independent from patient’s cardiac history.

### Caffeine levels and ischemic segments

Despite varying caffeine levels after similar caffeine intake (200 mg each) at our institution, no correlation could be found between caffeine levels and the number of involved ischemic segments. This is in line with Lee et al. [13], stating that the concentration of caffeine (at baseline or after supplementation) was not associated with percent defect reversibility. Furthermore, the amount of change of caffeine levels from the initial CMR to the second CMR exam after caffeine consumption had no effect on percent defect reversibility, *p* = 0.97. Reyes et al. [33] investigated 30 patients with known or suspected CAD with and without caffeine by clinically indicated myocardial perfusion imaging. They found that myocardial ischemia decreased by presence of caffeine with the standard use of 140 μg adenosine but did not change significantly with the use of the higher adenosine dose of 210 μg suggesting that in patients with prior caffeine consumption the protocol might be switched to the higher adenosine dose. The reason for this finding might be the competitive interaction between adenosine and caffeine, so receptor blockade by caffeine could be surmounted by an increased dose of adenosine. However, the higher dose is not approved for use in the United States in imaging [1].

### Limitations

Since this is a single-center study, potential center-specific bias cannot be excluded. Furthermore, the results of this study were raised in a population with extensive ischemic burden, and might not be transferred to patients which demonstrate an ischemic burden comprising only 1 or 2 myocardial segments. Therefore, our results cannot be generalized to all patients with CAD. Furthermore, quantification of ischemic burden by a 2D 3-slice approach may be inferior to a 3D full coverage approach. However, our 3-slice approach is common practice for clinical routine, and underlines the real-world character of this study.

We have not addressed the ingestion of different caffeine amounts in order to detect a potential threshold at which caffeine shows definite impact on the extent of ischemic burden. However, intention of our study was to reach significant serum levels of caffeine to demonstrate the influence of caffeine on myocardial ischemic burden. Furthermore, a previous study from Lee et al. [13] assessed adenosine-induced myocardial perfusion imaging defects over a broad range of caffeine concentrations with SPECT, and found no significant caffeine effect.

Moreover, most of the aforementioned studies are performed with SPECT since there is only scarce data about the effect of caffeine on adenosine stress CMR, which is known to have better spatial resolution than SPECT. Therefore, not all the data might be applied to the technique of CMR.

In this study, coronary angiography was used as the gold standard for the detection of significant CAD. Nevertheless, one should keep in mind that the sole anatomical presence of a stenosis does not always provide sufficient information regarding its hemodynamic relevance. Thus, functional assessment by intracoronary pressure wire (FFR) or intravascular ultrasound studies would have been highly desirable, but was not carried out in this study.

## Conclusions

In high-risk patients with prior caffeine intake, we found less ischemic burden on their adenosine stress CMR compared to their caffeine-naïve adenosine stress CMR study. Since these differences can be detected visually in a sample of only 30 patients in a statistically significant way, the impact of caffeine in CMR diagnostic and prognostic assessment cannot be regarded as negligible. Therefore, we recommend patients scheduled for adenosine stress CMR to refrain from caffeine in order to preserve 1) the high diagnostic accuracy of adenosine stress CMR for the detection of significant coronary stenosis, and 2) its high prognostic value which is related to the size of ischemic burden.

## Abbreviations

CAD: Coronary artery disease
CMR: Cardiovascular magnetic resonance
EF: Ejection fraction
IQR: Interquartile range
LGE: Late gadolinium enhancement
LV: Left ventricle/left ventricular
NYHA: New York Heart Association
SPECT: Single-photon emission computed tomography
